# Prospective radiotherapy quality Assurance leads to delineation guideline refinements for recurrent rectal cancer: Experience from the PelvEx II study

**DOI:** 10.1016/j.ctro.2025.100934

**Published:** 2025-02-13

**Authors:** F. Piqeur, B.J.P. Hupkens, D.M.J. Creemers, S. Nordkamp, M. Berbee, J. Buijsen, H.J.T. Rutten, C.A.M. Marijnen, J.W.A. Burger, H.M.U. Peulen

**Affiliations:** a Department of Radiation Oncology, Catharina Hospital, Michelangelolaan 2, 5623EJ Eindhoven, the Netherlands; b Department of Radiation Oncology, The Netherlands Cancer Institute, Plesmanlaan 121, 1066 CX Amsterdam, the Netherlands; c Department of Radiation Oncology, Leiden University Medical Centre, Albinusdreef 2, 2333ZA Leiden, the Netherlands; d Department of Radiation Oncology, MAASTRO, GROW School for Oncology and Reproduction, Maastricht University Medical Centre+, Doctor Tanslaan 12, 6229ET Maastricht, the Netherlands; e Department of Surgery, Catharina Hospital, Michelangelolaan 2, 5623EJ Eindhoven, the Netherlands; f GROW School of Oncology and Developmental Biology, University of Maastricht, Universiteitssingel 40, 6229ER Maastricht, the Netherlands

**Keywords:** Locally recurrent rectal cancer, Quality assurance, Delineation guideline

## Abstract

**Introduction:**

Target volume delineation in locally recurrent rectal cancer (LRRC) is clinically challenging. To ensure the quality of chemoradiotherapy (CRT) within the PelvEx II trial, a delineation guideline was developed and prospective quality assurance (QA) was instated for all patients. Guideline adherence, the impact of QA on target volumes, and subsequent guideline refinements are described in this paper.

**Methods and materials:**

All PelvEx II patients, either RT naive patients (50–50.4 Gy) or reirradiation (30 Gy) patients, were eligible for QA prior to CRT. An online meeting with the treating physician and the QA team was planned for each patient prior to treatment, to peer review delineations. Adherence to each of the 7 (reirradiation) or 8 (RT naive) guideline recommendations was scored. Suggested target volume adjustments and any reasons to deviate from protocol were noted. When applicable, target volumes before and after QA were compared. Possible protocol refinements were discussed amongst the trial QA team.

**Results:**

Prospective review of 113 cases of LRRC was performed, resulting in a high QA compliance rate of 90 %. All guideline recommendations were followed in 53 % of cases. Changes to the GTV and CTV were advised in 21 and 39 cases respectively. A median increase of GTV (+29 % (p < 0.001)) and CTV (+15 % (p < 0.001)) was seen in reirradiation patients, versus a median CTV increase of + 6 % (p = 0.002) in RT naive patients following QA. Deviations from protocol were accepted in 30 cases (27 %). Thirteen protocol refinements were agreed upon.

**Conclusion:**

Peer-review of LRRC target volumes leads to altered target volumes in up to 48% of cases, resulting in an updated delineation guideline.

## Highlights

• Real-time trial QA of target volume delineations in an online fashion yields a high QA compliance (90%).
• Peer-review of target volume delineations in LRRC results in alterations in half of discussed cases.
• Common reasons for non-compliance, protocol deviations and reoccurring questions were used to update the current guideline.

## Introduction

The incidence of locally recurrent rectal cancer (LRRC) has decreased due to the introduction of total mesorectal excision (TME) surgery and neoadjuvant (chemo)radiotherapy (nCRT) in the primary setting.[1], [2], [3], [4], [5] Patients who develop a local recurrence have a poor oncological outcome, with a 5-year overall survival of approximately 30 % in patients undergoing treatment with a curative intent.[3], [6], [7].

Treatment for patients with non-metastatic, (borderline) resectable LRRC in the Netherlands currently consists of nCRT, followed by surgery.[1], [3], [8], [9], [10] Depending on previous radiotherapy, either chemoradiotherapy (25x2Gy or 28x1.8 Gy) or chemo reirradiation is used (15x2Gy), both with concomitant capecitabine.[11], [12] The rationale of nCRT is to downsize tumour volume preoperatively, to facilitate a radical surgical resection (R0). To date, the R0 resection rate is the most commonly reported predictor of oncological outcome. Recently, achieving a pathological complete response (pCR) after neoadjuvant treatment has also been shown to be a strong predictor of oncological outcome.[13], [14] The use of induction chemotherapy before nCRT may lead to more patients achieving a pCR.[14], [15], [16] Therefore, the randomised PelvEx II trial was initiated, investigating the benefit of induction chemotherapy before nCRT and surgery for LRRC.[17].

Currently there are no international guidelines regarding target volume delineation (TVD) for LRRC, potentially introducing heterogeneity which in turn may even affect the ability to achieve an R0 resection. However, developing a guideline is complicated due to heterogeneity and low incidence of the disease. In LRRC, there is a loss of normal anatomical boundaries after surgery (most notably the mesorectal fascia), often leading to an extra-anatomical presentation of LRRC, such as against the pelvic wall or presacral. Moreover, there is a large variation in previous rectal cancer treatment (i.e., no neoadjuvant treatment, previous short-course or long-course (chemo)radiotherapy, use of induction or consolidation chemotherapy) further introducing heterogeneity. Lastly, prospective data that can aid in TVD is lacking, as only a handful of prospective trials for LRRC have been performed and data regarding LRRC treatment failure is scarce.

To overcome these issues, the PelvEx II trial aimed to standardize radiotherapy for LRRC. The first step was to develop a consensus-based delineation guideline, as published previously.[18] Secondly, it is important to monitor guideline adherence and guideline applicability, to improve the delineation guideline if necessary. A prospective Quality Assurance (QA) programme was therefore instated, consisting of peer-review of TVD.

The aim of this study is to assess the impact of a prospective QA programme within the PelvEx II trial, regarding delineation guideline adherence and applicability, and to refine the current delineation guideline if and where necessary.

## Materials and methods

The PelvEx II trial opened in November 2020. The trial includes patients with non-metastatic, (borderline) resectable LRRC after a partial or total mesorectal excision for primary rectal or distal sigmoidal cancer.[17] LRRC staging is performed according to the PelvEx II radiology standard operating procedure, including a description of all (potentially) involved organs and structures. Patients are randomized to either the standard arm, consisting of nCRT followed by surgery, or to the experimental arm, consisting of induction chemotherapy (either 3–4 cycles of CAPOX or 4–6 cycles of FOLFOX or FOLFIRI), followed by nCRT and surgery. All trial patients receive nCRT, either 25x2Gy or 28x1.8 Gy for RT naive patients, or 15x2Gy for patients undergoing reirradiation, with concomitant capecitabine, depending on primary rectal cancer treatment.

A PelvEx II delineation protocol was established during several multidisciplinary consensus meetings with LRRC experts from the Netherlands and Sweden, as described previously.[18] In the PelvEx II trial, all radiation oncologists were asked to delineate according to the delineation guideline.

Prospective review of all delineations was instated for all randomized in the PelvEx II trial patients from 01 to 05-2021 onwards, as there was no coordinating investigator available prior to this date. The QA programme consisted of an online peer-review meeting with the treating radiation oncologist, at least one member of the PelvEx II QA team (HP, JB, MB, BH, CM) and the coordinating investigator (FP, DC). A meeting was planned as soon as possible after delineation was performed, to avoid treatment delay. Patients treated in QA centres (i.e., Maastro and Catharina Hospital Eindhoven) were internally reviewed by QA team members.

During the QA meeting, the treating physician presented the case, including information on primary and recurrent disease. GTV and CTV delineations were evaluated. PTV margins are dependent on local protocol and were therefore not reviewed in QA meetings but should be between 5 and 10 mm, as stated in the guideline.

Adherence to each recommendation was scored in a binary fashion (i.e., compliant yes/no). Suggested target volume alterations were noted, and radiation oncologists were asked to save all original target volumes (pre-QA), as well as altered target volumes (post-QA), if applicable. Any agreed upon protocol deviations were noted with the discussed considerations, as were any questions that arose from the instated protocol. Radiotherapy DICOM data were collected after radiotherapy was completed.

All patients randomized by December 31, 2023, were selected for analysis. Patients were excluded in case of withdrawal of informed consent, screening failure, or failure to start nCRT. Median GTV and CTV were calculated and compared before and after QA, when applicable.

Categorical data is shown as an absolute number with percentages. Continuous data is either reported as a mean or median, as fit, with a standard deviation (SD) or interquartile range (IQR) respectively. Categorical comparisons are performed using the Chi-Square and Fisher exact test, as applicable. Continuous data are compared by the Mann-Whitney *U* test and the Wilcoxon Signed Rank test, as applicable. Two-sided p-values of < 0.05 were deemed statistically significant for all performed analyses. Statistical analyses were performed using IBM SPSS Statistics for Windows, Version 29.0, IBM Corp. Released 2022. Armonk, NY: IBM Corp.

Propositions for guideline refinement were written by the coordinating investigator following data analysis, incorporating the most common reasons for non-adherence, protocol deviations and questions that arose from QA meetings. Propositions were sent to all members of the QA team and discussed in a guideline meeting. A refined protocol was then written and approved by all members of the PelvEx II QA team.

## Results

**Fig. 1:**
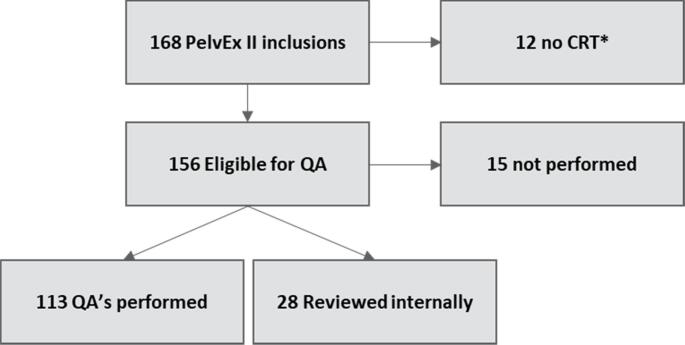
Flowchart of all PelvEx II patients randomized before 31–12-2023 that were eligible for Quality Assurance meetings. An overall QA compliance rate of 90% (141/156) of patients eligible for QA were reviewed. *CRT not performed due to withdrawal of informed consent, screening failures or progressive disease.

A total of 168 patients were randomized by December 31, 2023. 12 patients were not eligible for nCRT and therefore excluded from analysis (Fig. 1). Overall, 141/156 patients (90 %) received peer review. QA was performed in 21 trial centres. The number of QA meetings per centre ranged from 1 to 12, with a median of 3. The baseline characteristics of all patients eligible for QA are shown in Table 1.

**Table 1:** Baseline characteristics of all patients eligible for QA (n = 156).

**Baseline characteristics**		**n**	**%**
**Primary tumour**			
**Gender**	Male	113	72.4 %
	Female	43	27.6 %
**cT-stage**	cT0-2	29	21.6 %
	cT3-T4	105	78.4 %
**cN-stage**	cN0	46	34.8 %
	cN1-2	86	65.2 %
**Neoadjuvant treatment**	None	63	40.4 %
	CRT	52	33.3 %
	Chemotherapy + CRT	10	6.4 %
	5x5 Gy	18	11.5 %
	Chemotherapy + 5x5 Gy	13	8.3 %
**Surgical procedure**	LAR	71	45.8 %
	APR	56	36.1 %
	TaTME*	15	9.7 %
	Recto sigmoidal resection	13	8.4 %
Any metastases before LRRC diagnosis**	Yes	30	19.2 %
**Local recurrence**			
**Age at diagnosis**	Mean (SD)	64	9.9
**Time from surgery to LRRC diagnosis (months)**	Median (IQR)	23	12–39
**Multifocal recurrence**	No	126	80.8 %
	Yes	30	19.2 %
**Type of recurrence**	Mucinous	28	19.0 %
	Solid	110	74.8 %
	Fibrotic	5	3.4 %
	Other	4	2.7 %
**Involved compartments**	Anterior	50	32.7 %
	Posterior	70	45.8 %
	Lateral	86	56.2 %
	Central	61	39.9 %
**Size (largest) lesion (mm)**	Median (IQR)	32	20–54

Missing data was excluded from analysis.

*TaTME: Transanal total mesorectal excision.

**Metastases at LRRC diagnosis and/or within 6 months prior to diagnosis is an exclusion criterium. Any metastases before LRRC diagnosis had to be diagnosed and treated at least 6 months prior to diagnosis of the local recurrence for patients to be eligible for the PelvEx II trial.

External peer review was performed in 113 patients (72 %), of which 64 were undergoing reirradiation (57 %), and 49 were RT naive (43 %). In an additional 28 patients, internal QA was performed. In the remaining 15 patients QA was not performed because the QA programme had not yet started (n = 5) (i.e., before 01–05-2021), or because QA was not possible due to time constraints or lack of response (n = 10).

Internally peer-reviewed cases were excluded from analyses as it was not systematically recorded whether changes to target volumes were made. Of the 113 cases that underwent external peer-review, DICOM data was retrieved for 101 cases (89 %). Data could not be retrieved for 12 cases as patients had not yet finished CRT (n = 6), or there was no response to the call for data (n = 6).

There were seven guideline recommendations for reirradiation, and eight for RT naive patients, as summarized in table 2. All guideline recommendations were followed in a total of 60/113 cases (53 %). In reirradiation patients, 58 % of cases were completely guideline compliant (7/7 recommendations followed), versus 47 % in RT naive patients (8/8 followed). Nineteen reirradiation cases deviated from one recommendation (30 %), seven from two recommendations (11 %) and one from three recommendations (1 %). Twelve RT naive cases deviated from one recommendation (25 %), eleven from two recommendations (22 %) and three from three recommendations (6 %).

The most common reasons for non-compliance were editing towards OAR (n = 28), not incorporating all fibrosis into target volumes (n = 11) and not delineating all elective target volumes (n = 6) or remaining mesorectal fat (n = 8) in RT naive patients, despite the protocol stating that elective target volumes should be delineated as in locally advanced rectal cancer (LARC). An overview of compliance to each recommendation can be found in Table 2.

**Table 2:** Summary of recommendations in the PelvEx II delineation guideline. The percentage of peer-reviewed cases adhering to each original recommendation is shown for reirradiation patients (receiving 15x2Gy) and RT-naive patients (receiving either 25x2Gy or 28x1.8 Gy).

	**Guideline recommendation**	**Reirradiation (n = 64)**	**RT naive (n = 49)**
1	GTV was correctly identified	58 (91 %)	48 (98 %)
2	All (pre-chemo) GTV was incorporated in the CTV	64 (100 %)	47 (96 %)
3	Complete fibrosis (when applicable) was covered by GTV or CTV	**54 (84 %)**	48 (98 %)
4	A correct margin of at least one cm was used to CTV	62 (97 %)	43 (90 %)
5	All GTV lesions were incorporated in one CTV with correct margins	63 (98 %)	47 (96 %)
6	CTV was not edited towards organs at risk	**52 (83 %)**	**33 (67 %)**
7a	No elective target volumes were delineated when performing reirradiation	60 (94 %)	n.a.
7b	Elective target volumes were delineated as in LARC	n.a.	43 (88 %)
8	All (remaining) mesorectal fat was delineated as in LARC	n.a.	41 (84 %)

Changes to target volumes were made in 54 cases. Pre-QA data was not saved and therefore not retrievable in 8/54 patients.

**Fig. 2:**
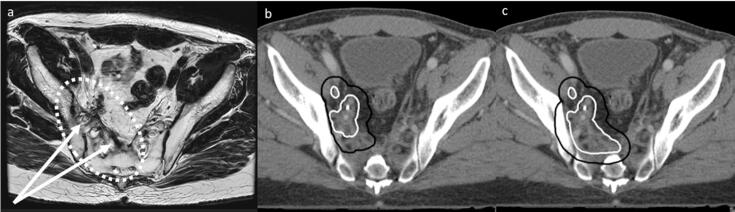
Example of an alteration made during a QA meeting. From left to right, the pre-treatment diagnostic MRI (a), planning CT with pre-QA target volumes (b) and planning CT with post-QA target volumes (c) are shown. The GTV is shown in white, the CTV in black. The original GTV was extended following QA to encompass the complete fibrosis. The CTV was originally edited out of the bone, but it was advised not to do so given the invasive nature of the disease and the risk of an R1-resection at the lateral side wall.

Changes to the GTV were advised in 21 cases, of which 20 led to an increase in GTV volume. In 11/21 cases, not all fibrosis was incorporated into target volumes, as shown in Fig. 2 (n = 10 reirradiation, n = 1 RT naive). Other reasons for proposed GTV editing included but were not exclusively; incorporating the whole circumference in the GTV in an intraluminal recurrence (n = 1), expanding the cranio-caudal margins in an intraluminal recurrence (n = 1), incorporating an adjacent fistula within the GTV (n = 1), incorporating the previous anastomosis into the GTV in an intraluminal recurrence (n = 1), and extending the GTV due to uncertainty of image registration (n = 2).

A statistically significant increase in median GTV is seen in reirradiation patients where alterations were advised (n = 15, +29 %, p < 0.001), but the observed increase does not reach significance in RT naive patients, most likely due to low numbers (n = 3, +36 %, p = 0.109) (Table 3). Notably, post-QA GTV was significantly larger in patients undergoing reirradiation (58 cc) compared to RT naive patients (33 cc) (p = 0.048).

**Table 3:** Overview of number of changes made, protocol deviations, and median gross target volume (GTV) and clinical target volume (CTV) for reirradiation and RT naive patients.

		**Reirradiation (n = 64)**		**RT naive (n = 49)**		**All (n = 113)**		**p-value**
		**n=**	**%**	**n=**	**%**	**n=**	**%**	
**Any changes made**	Yes	30	47	24	49	54	48	0.824
	No	34	53	25	51	59	52	
**Number of changes**	1	26	87	17	71	43	80	
	2	4	13	6	25	10	19	
	3	0	0	1	4	1	2	
**Protocol deviation**	Yes	15	23	15	31	30	27	0.394
**Volumes (n = 101)**		**Reirradiation (n = 60)**		**RT naive (n = 41)**		**Total (n = 101)**		**p-value**
		**Median**	**IQR**	**Median**	**IQR**	**Median**	**IQR**	
**GTV**	cc	58	16–119	33	16–59	48	16–102	0.048
**CTV**	cc	275	148–478	464	365–611	374	214–553	<0.001

Changes to the CTV were advised in 39 cases, leading to an increase in CTV volume in 36 cases. The most common advice was to reverse any editing towards OAR (n = 16) (Fig. 2), most notably the sacrum, and to adjust delineated elective volumes (n = 16). In 7 cases, this adjustment consisted of extending the elective CTV to encompass the obturator nodes (n = 7).

CTV was significantly larger in RT naive patients compared to reirradiation patients, due to elective target volumes (Table 3). A statistically significant increase in median CTV is seen in both reirradiation and RT naive patients following QA (reirradiation n = 24, +15 %, p < 0.001; RT naive n = 14, +6%, p = 0.002). A statistically significant increase in median PTV is also seen in reirradiation and RT naive patients (respectively + 15 % (n = 20, p < 0.001) and + 3 % (n = 10, 0.009). In 21 of the 30 cases (70 %) in which before and after CTV and PTV volumes were available, the old PTV did not encompass the new CTV, implying that the changes made during QA were of significance to the final treatment in a majority of redacted cases.

Protocol deviations were accepted in 30 cases (27 %) (Table 3). The most common reasons for protocol deviations were reducing elective target volumes because of potential toxicity (n = 11) (in cases where risk of toxicity was deemed high, such as significant small bowel within the pelvis, and oncological safety was not deemed compromised) and extending the CTV beyond what is currently defined in the protocol (n = 12), for example for better coverage of expected resection margins.

In 22 cases, the instated protocol did not give enough guidance on how to delineate. In 6 instances, questions were raised regarding editing towards OAR, mainly towards pelvic bones. Three questions were posed regarding intraluminal recurrences, asking whether or not to delineate the whole circumference as GTV and which cranial-caudal margins should be used (i.e., 1 or 2 cm). The last group of questions was only applicable to RT naive patients, namely, which LARC delineation recommendations should and should not be followed; is it necessary to delineate up to S1 in case of perineal recurrences and is it necessary to delineate all elective fields in case of a high nodal recurrence after PME for distal sigmoidal cancer.

During the guideline meeting, thirteen guideline adjustments were adopted by the QA team. The updated delineation guideline can be found in the supplementary material and is summarized in Table 4.

**Table 4:** Summary of original or new (in bold) GTV and CTV recommendations for LRRC. Exceptions and clarifications are provided in the full delineation guideline in the supplementary material.

GTV	• Delineate all macroscopically visible tumour in primary staging • Delineate only visibly involved areas of surrounding organs • Consult with a dedicated radiologist to determine the extent of the GTV • In case of induction chemotherapy: • If major regression occurs, adjustment towards other non-involved structures is allowed • If a complete response is suspected, GTV should still be defined (i.e., remaining tumour bed or fibrosis) • **If the tumour is located in fibrosis, the whole fibrosis should be incorporated** • **If the tumour is located within an abscess, the whole abscess should be incorporated** • **In case of an intraluminal recurrence, the whole circumference of the lumen should be incorporated**
CTV	• Consult with the treating surgeon to determine surgical resection margins at risk for irradical resection and discuss planned type of surgery • CTV = GTV + 1 cm margin • Combine multifocal recurrences into one CTV using logical anatomical boundaries and staying at least 1 cm beyond the GTV in all directions • Do not adjust CTV towards other organs • If applicable, all pre chemotherapy GTV must be included in the CTV • **In case of an intraluminal recurrence, a cranio-caudal border of 2 cm beyond the GTV is advised and a circumferential margin of 1 cm is advised** • In presacral recurrences, extending the CTV towards the lateral sidewall should be considered, to ensure coverage of surgical resection margins • In reirradiation patients, no elective nodal delineation is advised • In RT naive patients, elective target volumes should always be delineated • **Remaining mesorectal fat and nodes, presacral nodes, internal iliac nodes and obturator nodes should always be included** • **The cranial border of the CTV is at least the bifurcation of the common iliac arteries into the external and internal iliac artery/sacral promontory, with allowance for national adjustments, or one centimeter above the most cranial GTV if it extends beyond the bifurcation.** • **In case of involvement, delineation of the abdominal presacral lymph nodes and external iliac lymph nodes should be considered** • **The ischiorectal fossa and sphincter complex should be incorporated in case of involvement or a perineal recurrence**

## Discussion

In this study, we describe our experience implementing the first formal delineation guideline for LRRC, and subsequent peer review of delineations within the PelvEx II trial. For trial QA, we use a simple, online approach, minimizing time investment (+/-15 min), and omitting the need for data transfers, resulting in a high QA participation (90 %). By organizing a meeting as soon as TVD has been performed and changing target volumes in real time, we avoid treatment delay. We show that alterations are recommended in 48 % of cases, clearly highlighting the need for prospective peer review in LRRC. As protocol deviations are reported in 27 % of cases, the instated protocol has been tailored to improve guideline adherence.

Based on the presented data, several lessons can be learned. The most common reason for non-compliance was editing towards OAR. We currently advise not to edit the CTV towards OAR, as there is a loss of anatomical boundaries in LRRC after surgery, which may lead to more invasion of surrounding structures, potentially hampering surgical resection. Moreover, the positive predictive value of MRI may be limited for tumour invasion into surrounding structures, even though it is the gold standard modality.[8], [9], [10], [19], [20], [21], [22] One exception within the guideline was that editing towards pelvic bones is acceptable if it is highly likely that there is no bony invasion (i.e., no invasion of the lateral side wall in case of a presacral recurrence). This was often interpreted as that editing towards bony structures is acceptable when it is not described as invaded on MRI. Consequently, editing towards pelvic bones was seen in numerous cases and recommended against during QA. In the revised guideline, this recommendation has been reformulated.

A common QA recommendation is to extend the GTV to encompass all surrounding fibrosis within the GTV. It was previously recommended to delineate all fibrosis as GTV if the difference between tumour and fibrosis was unclear. In cases where the distinction was clear, fibrosis should at least be covered by the CTV. In clinical practice, PET-positive areas within fibrosis were often deemed GTV and therefore surrounding fibrosis was deemed CTV. Although this is guideline compliant, the potential risk of false negativity of the PET-CT is often underestimated, and underestimation of GTV should be avoided.[21] Therefore, it was agreed that surrounding fibrosis should always be incorporated in the GTV, regardless of the PET-CT, leading to a guideline adaptation.

The guideline stated that RT naive LRRC cases should be delineated as if they were LARC. However, a lot of protocol deviations regarding elective target volumes in RT naive patients were reported, implying this recommendation was not sufficiently detailed. It was therefore proposed to explicitly specify which elective volumes should be irradiated. Each LRRC should be deemed at very high risk for locoregional failure due to the loss of anatomical boundaries and the reported poor LRFS.[23] Therefore, all remaining mesorectal fat, all (remaining) mesorectal nodes, presacral nodes, internal iliac nodes and obturator nodes should be irradiated. Other elective volumes should only be delineated in specific cases, as stated within the full guideline.

Lastly, tailoring the protocol to different recurrence types seen within the trial seemed necessary, based on reoccurring questions during QA. These questions led to additional recommendations for tumours located intraluminal, tumours located in an abscess, very high recurrences (completely above S2/S3) and very distal recurrences.

As of now, the updated and approved delineation guideline has been distributed amongst PelvEx II trial centres. Ideally, the updated guideline would lead to more homogeneity of TVD within the trial. Refining the guideline may have the added benefit of decreasing the number of modifications needed, as seen in primary rectal cancer, where two central review platform studies showed that refinement of recommendations led to a significant decrease in the number of CTV modifications.[24], [25].

Interestingly, the study group was also able to show a learning effect after ten performed reviews, albeit in primary cancer.[25] A learning effect cannot be discerned yet based on the current QA data, which may be explained by the heterogeneity and complexity of LRRC, or by the fact that exposure to LRRC is often limited for individual physicians.

A recent review by Brooks et al. proposes prospective review for all trial patients, as on-trial protocol deviations are found continuously, even after benchmark accreditation across several tumour types.[26] It is therefore reasonable to assume that even if a learning effect did occur, this may not be sustained in LRRC. They further advocate a risk-adjusted approach, tailoring the intensity of QA to trial-specific factors. Although planning is relatively simple for LRRC given there are no formal dose constraints, TVD is highly complex. Therefore, the QA programme will be continued until all patients have undergone nCRT. Based on accrual so far, this would be expected in 2027. A learning effect will be re-evaluated based on available data.

Given the number of alterations made within the trial, it is likely that a peer review process for all patients with LRRC would be beneficial, as seen in studies investigating peer review in non-trial populations.[27] By doing so, all patients could benefit from research developments and QA in real time. An additional benefit would be that the current guideline can be tested for applicability in a palliative population, as up to 50 % of patients with LRRC present with either metastatic or local irresectable disease.[6], [7], [28] The guideline was initially developed for the curative setting and is now ongoingly tested within a curative population. For a palliative population, other outcomes such as quality of life and local control may be more important than tumour volume downstaging. Therefore, prospective peer-review of all LRRC cases may help answer questions in regard to TVD for a palliative population.

To summarize, prospective peer review of LRRC target volumes leads to changes in 48 % of cases, highlighting the need for collaboration in this complex tumour type. Based on the current data, the protocol was further refined by incorporating commonly recommended alterations, tailoring recommendations to different tumour types and incorporating frequent protocol deviations.

Several limitations do apply. Although the use of this delineation guideline will likely result in more consistent TVD throughout the trial, there is no gold standard to determine the accuracy of delineations. The guideline was developed based on consensus with multidisciplinary experts in the Netherlands and Sweden, but prospective data to substantiate the validity of the protocol is still lacking. Lastly, no information on response to chemotherapy can be disclosed as the trial is ongoing, but it may have an effect on how delineations are performed.

## Conclusion

Peer review of target volume delineations after guideline implementation for LRRC led to alterations in 48 % of cases, highlighting the need for real-time QA of LRRC delineations. Based on advised alterations, protocol deviations, and reoccurring questions, the guideline was updated. By continuing trial QA, development of an evidence-based delineation guideline will be possible.

## CRediT authorship contribution statement

**F. Piqeur:** Conceptualization, Data curation, Formal analysis, Investigation, Methodology, Writing – original draft. **B.J.P. Hupkens:** Conceptualization, Investigation, Writing – review & editing. **D.M.J. Creemers:** Investigation, Writing – review & editing. **S. Nordkamp:** Investigation, Writing – review & editing. **M. Berbee:** Conceptualization, Investigation, Writing – review & editing. **J. Buijsen:** Conceptualization, Investigation, Writing – review & editing. **H.J.T. Rutten:** Conceptualization, Methodology, Supervision, Writing – review & editing. **C.A.M. Marijnen:** Conceptualization, Methodology, Supervision, Writing – review & editing. **J.W.A. Burger:** Conceptualization, Funding acquisition, Project administration, Supervision, Writing – review & editing. **H.M.U. Peulen:** Conceptualization, Investigation, Methodology, Project administration, Resources, Supervision, Writing – review & editing.

## Declaration of Competing Interest

The authors declare that they have no known competing financial interests or personal relationships that could have appeared to influence the work reported in this paper.
