# Supplementary material for: Prospective radiotherapy quality Assurance leads to delineation guideline refinements for recurrent rectal cancer: Experience from the PelvEx II study
Source: Clin Transl Radiat Oncol. 2025 Feb 13;52:100934. doi: 10.1016/j.ctro.2025.100934 (PMC11894322; doi:10.1016/j.ctro.2025.100934)
Supplement: Supplementary Data 1 [file mmc1.docx]

**Supplementary material**

**Table S1.** Percentage of peer-reviewed cases adhering to each original recommendation after QA for reirradiation patients (receiving 15x2Gy) and RT-naive patients (receiving either 25x2Gy or 28x1.8Gy).

|  | **Guideline recommendation** | **Reirradiation (n=64)** | **RT naive (n=49)** |
| --- | --- | --- | --- |
| 1 | GTV was correctly identified | 62 (97%) | 49 (100%) |
| 2 | All (pre-chemo) GTV was incorporated in the CTV | 64 (100%) | 49 (100%) |
| 3 | Complete fibrosis (when applicable) was covered by GTV or CTV | **61 (95%)** | 49 (100%) |
| 4 | A correct margin of at least one cm was used to CTV | 63 (98%) | 48 (98%) |
| 5 | All GTV lesions were incorporated in one CTV with correct margins | 63 (98%) | 49 (100%) |
| 6 | CTV was not edited towards organs at risk | **59 (92%)** | **43 (88%)** |
| 7a | No elective target volumes were delineated when performing reirradiation | 62 (97%) | n.a. |
| 7b | Elective target volumes were delineated as in LARC | n.a. | 46 (94%) |
| 8 | All (remaining) mesorectal fat was delineated as in LARC | n.a. | 45 (92%) |

**PelvEx II**

Chemoradiotherapy manual

**TABLE OF CONTENTS**

1. Purpose and scope 3

2. QA Meeting 4

3. Radiotherapy treatment planning 5

4. Patient set-up 5

5. Patient data acquisition 5

6. Use of contrast 5

7. Target volumes 6

7.1 Gross tumour volume (GTV) 6

7.2 Clinical target volume (CTV) 9

7.2.1 Patients undergoing reirradiation 10

7.2.2 RT-naïve patients 12

7.3 Planning target volume (PTV) 13

8. Dose and volume guidelines 13

8.1 Radiotherapy-naïve patients 13

8.2 Previously irradiated patients 13

8.3 Target dose constraints 13

8.4 Organs at risk 14

9. Treatment planning 14

10. On treatment verification 14

11. Concomitant chemotherapy 15

12. Minimal requirements for chemoradiotherapy reporting 15

13. Contact details 16

14. References 17

# Purpose and scope

This document has been developed for and applies to the PelvEx II study and serves as a guideline for the process of planning and delivering (chemo)radiotherapy treatment in radiotherapy-naive and previously irradiated patients with locally recurrent rectal cancer enrolled in the study. The aim of this document is to ensure uniform delivery of radiotherapy and the collection of high-quality data for study analysis.

As recognized in the first version of this SOP, variability in target volumes can occur. Four multidisciplinary delineation workshops with PelvEx II trial centres in the Netherlands and Sweden were organized to overcome this issue. In these workshops, consensus was reached by participating radiation oncologists, but also surgeons and an expert radiologist, on how to define target volumes for our trial population. The development of the delineation guideline has been published by Piqeur and Hupkens et al.^1^ Following the development of the delineation guideline, a real-time quality assurance (QA) programme has been instated, as large inter-observer variation was reported.

The initial proposal was to perform a central review of the first 5 patients per centre. Based on preliminary data from the QA programme, of the first 168 trial patients, changes are made in up to 48% of cases after peer-review.^2^ Therefore, the QA programme will be continued for all patients included in the PelvEx II study.

Preliminary data further showed that consensus was reached to deviate from protocol in up to 30% of cases, and the protocol was deemed unclear in up to 22% of cases, when using SOP version 1.1 and 1.2.^2^ Therefore, recommendations in this SOP (version 2) have been refined and updated based on the observations made within the QA programme so far.

It is recognised that during the conduct of the trial, it may be necessary to further modify the defined protocol either because of the publication of new data regarding target volume definition, consensus views derived from radiotherapy planning workshops or observations during trial QA (see below). It is likely that any changes will be minor and can be introduced through modification of this radiotherapy planning document as they will not interfere with the key primary and secondary end points of the study. In the event of the need for a major change requiring alteration of the main protocol, a formal protocol amendment will be initiated.

To evaluate late effects of reirradiation, information on the previously given radiotherapy is essential. However, this may not be easy to retrieve. When available, the DICOM data of the previous treatment plan should be uploaded together with the reirradiation. If this is not available, the same volume parameters as for the re-irradiation plan should preferably be reported.

# QA Meeting

Participating physicians are asked to join a QA meeting before starting neoadjuvant chemoradiotherapy for their patient. The QA meeting is planned online via MS-teams by the trial coordinator. Ideally, the QA meeting is planned after the delineation has been made, but before any treatment planning is performed. The treating physician is asked to give a short introduction on the case, including information of primary rectal cancer presentation and treatment, and recurrent rectal cancer presentation. The physician is then asked to share their screen and show the performed delineations. Only target volumes are discussed. Delineation of OAR and treatment planning are not discussed. Radiation oncologists are asked to save all RT-structures. If any alterations are advised, this means old and new versions of the target volumes should be saved (for example: GTV_old and GTV_new).

Once patients have finished nCRT, all information on delineation and treatment planning is retrieved by the coordinating investigator. This information consists of the DICOM files of the planning CT, RT-structure, RT-dose, and RT-plan. The retained information is used to aid in further development of a delineation guideline for locally recurrent rectal cancer.

# Radiotherapy treatment planning

The use of a planning CT scan with target volumes delineated on each slice and pixel-based inhomogeneity correction is considered standard practice and is a mandatory requirement. Matching with the pre-treatment MRI is strongly recommended. Alternatively, delineation and planning on an MRI is allowed.

# Patient set-up

It is recommended that appropriate immobilisation and a scan/treatment position is used with which the site is familiar with. The supine position is recommended to improve reproducibility of the treatment position, but it is not mandated. A belly board may be used for treatments in the prone position, but it is not a requirement.

# Patient data acquisition

The scan limits are the superior aspect of L5 superiorly to 4 cm below the anal verge. The recommended slice thickness is 3 mm.

# Use of contrast

The use of intravenous and oral contrast is optional and should align with local centre policy.

# Target volumes

The target volume definition process requires the delineation of gross, clinical and

planning target volumes. These are defined below.

## Gross tumour volume (GTV)

All macroscopic visible must be delineated. This information is derived from the diagnostic imaging (at least a pelvic MRI, if available a PET-CT) and supplemented by clinical examination and possible endoscopic findings. Only involved areas of any organ will be included (i.e., it is not necessary to encompass the full bladder or vagina at involved levels).

If patients have received induction chemotherapy, all pre-chemotherapy macroscopic tumour should be delineated. Adjustment towards other structures is allowed in case of regression. In case of a complete response there will still be a GTV (the tumour bed/fibrosis after induction chemotherapy).

If the tumour is located in fibrosis, all adjacent fibrosis should be incorporated in the GTV.

**Reasoning***:* *Interpretation of radiological imaging of tumours in fibrosis can vary significantly, as shown in figure 1. Even if PET-positive areas within fibrosis seem to be distinguishable as tumour compared to surrounding fibrosis, it is advised to encompass all fibrosis in GTV, as shown in figure 2, given the risk of false negativity of the MRI and the PET-CT.*^3^


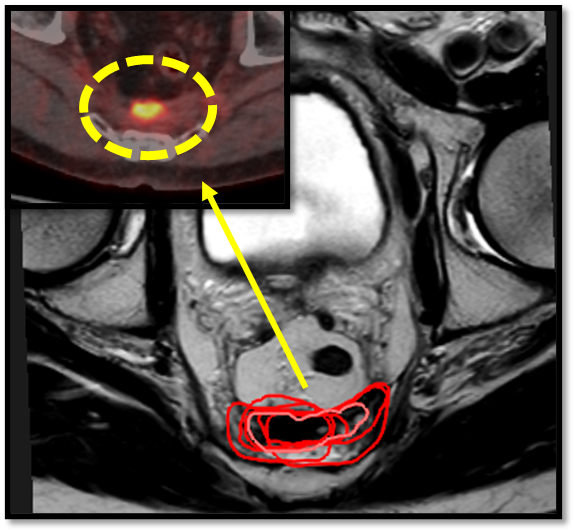
**Figure 1**: Delineation of a PET-positive tumour located in fibrosis. A (not yet published) delineation study showed differences in GTV interpretation in tumours located in fibrosis. 3/8 radiologists and 7/11 radiation oncologists delineated all fibrosis as tumour, whereas 5/8 radiologists and 4/11 radiation oncologists only delineated the PET-positive area within fibrosis as tumour. Consensus has been reached that all surrounding fibrosis should be deemed GTV, as shown in figure 2.


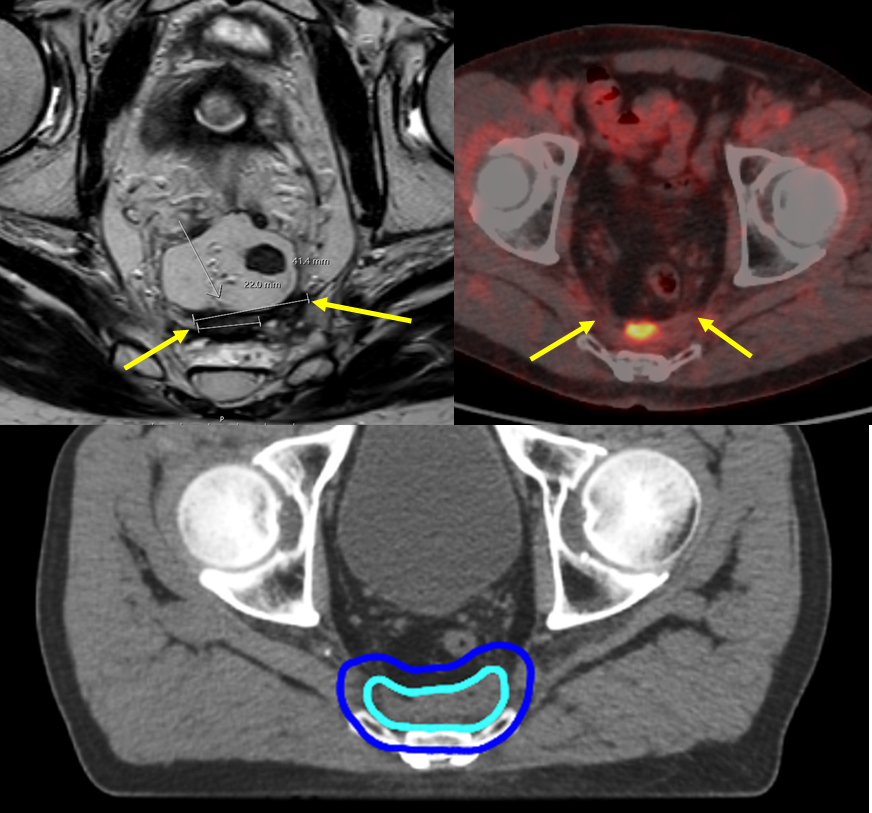


**Figure 2:** Example of how to delineate a presacral, fibrotic tumour. The GTV (light blue) should include all surrounding fibrosis. The CTV (dark blue) can then be extended towards the lateral sidewalls to guarantee that surgical resection margins are completely incorporated in the CTV. The CTV should not be edited towards the sacrum, as this area would be where a potential irradical resection may occur.

If the tumour is located within an abscess, the whole abscess should incorporated in the GTV.

**Reasoning**: In tumours located within an abscess, it is intuitive to account for potential tumour spread within circulating fluids by including the whole abscess within the GTV.

In case of an intraluminal recurrence (for example at the previous anastomosis), the whole circumference of the lumen should be deemed GTV.


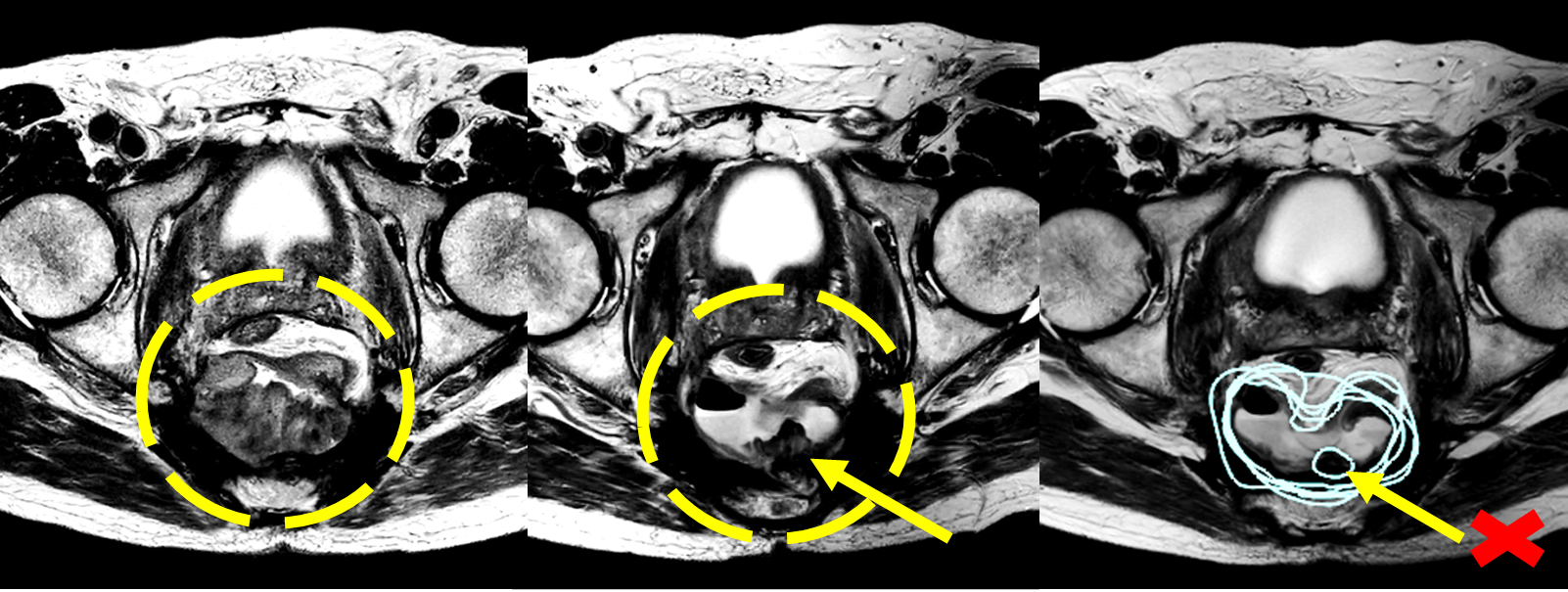


**Figure 3:** Example of a recurrence located in an abscess, before (left) and after (middle) induction chemotherapy. The whole remaining abscess should be delineated as GTV as shown on the right, instead of delineating only remnant solid tumour as GTV.


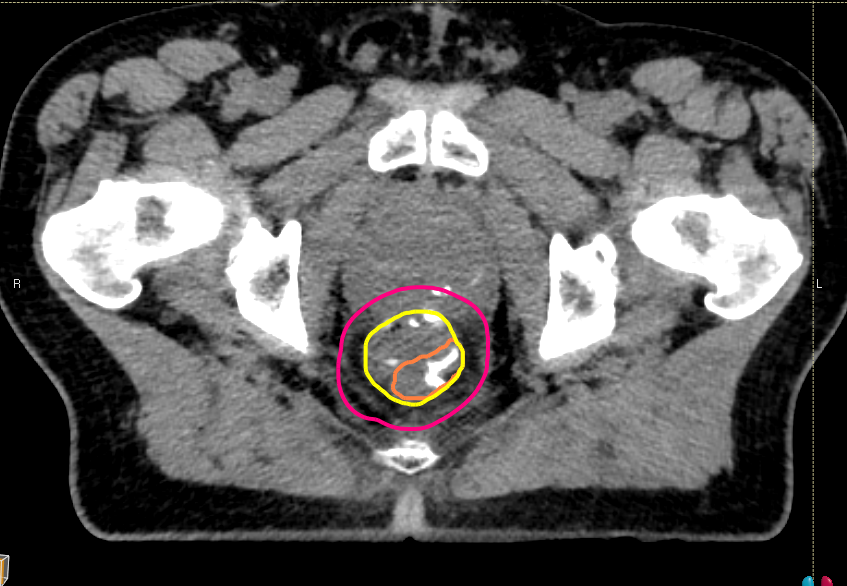


**Figure 4:** An example of an anastomotic recurrence previously reviewed during Quality Assurance. Consensus was reached to extend the original GTV (orange) to encompass the whole lumen (yellow).

##
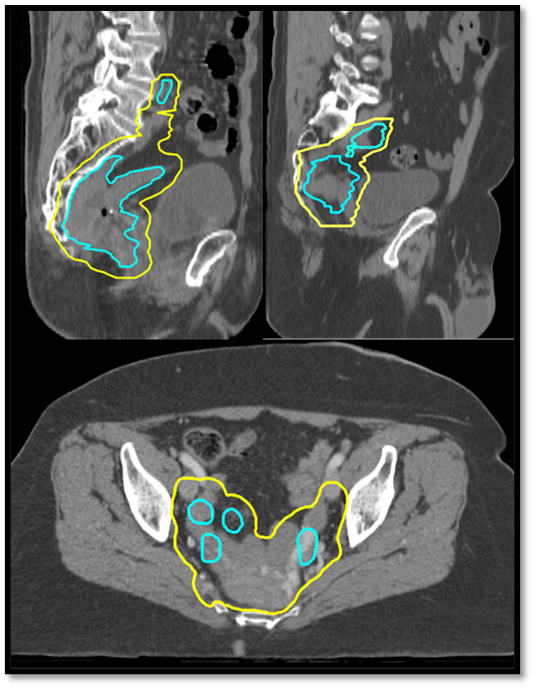
Clinical target volume (CTV)

The CTV should always encompass the GTV with at least a 1cm margin in all directions, irrespective of any additional elective volumes.

In case of multifocal recurrences, all localizations should be combined in one CTV, with logical anatomic boundaries. Small separate islands are not allowed. The limit of the CTV should be at least one centimeter beyond each GTV in every direction.

In case of intraluminal recurrences, a cranio-caudal border of 2 cm beyond the GTV is advised. Circumferentially, a one-centimeter margin is recommended, as in other recurrence types.

**Figure 5**: Three examples of multifocal recurrences. All GTV lesions (light blue) should be incorporated into one CTV with logical anatomical boundaries, irrespective of additional elective target volumes.

In case of a recurrence located in a rectal stump, delineation of the remaining rectal stump as CTV can be considered. A cranial border extending beyond the rectal stump can be adjusted to a minimum of 1 cm instead of 2 cm.

In case of presacral recurrences, extending the CTV margins towards the lateral sidewall to encompass complete surgical resection margins should be considered (figure 2, 6). In general, the advice is to rather be too extensive than too conservative when incorporating surgical resection margins within the CTV.

**
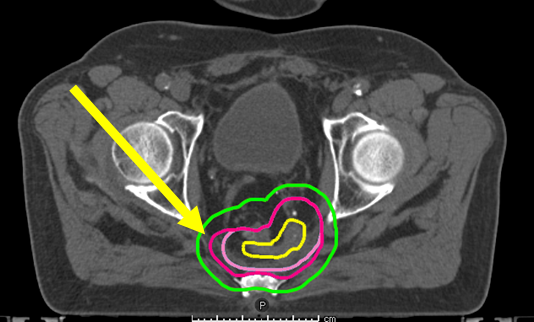
**

**Figure 6:** QA example in which the original CTV (light pink) was extended beyond the prescribed 1 cm margin, to ensure that surgical resection margins near the lateral side wall were adequately incorporated within the CTV (dark pink).

Adjustment of the CTV towards other organs is not allowed. This means the CTV often extends into surrounding organs and structures (such as bone, bladder, prostate, uterus).

In cases where it is beyond any clinical doubt whether or not surrounding organs are involved (for example when there are several non-involved structures laying between the OAR and the tumour), adjusting towards OAR can be considered.

**Reasoning**: Given the recurrent nature of the tumour and the loss of anatomical boundaries due to previous surgery, it is often at the border of structures and organs near the recurrence that surgeons are afraid an R1-resection may occur (figure 7). Adequate target coverage should therefore outweigh toxicity concerns in these areas.


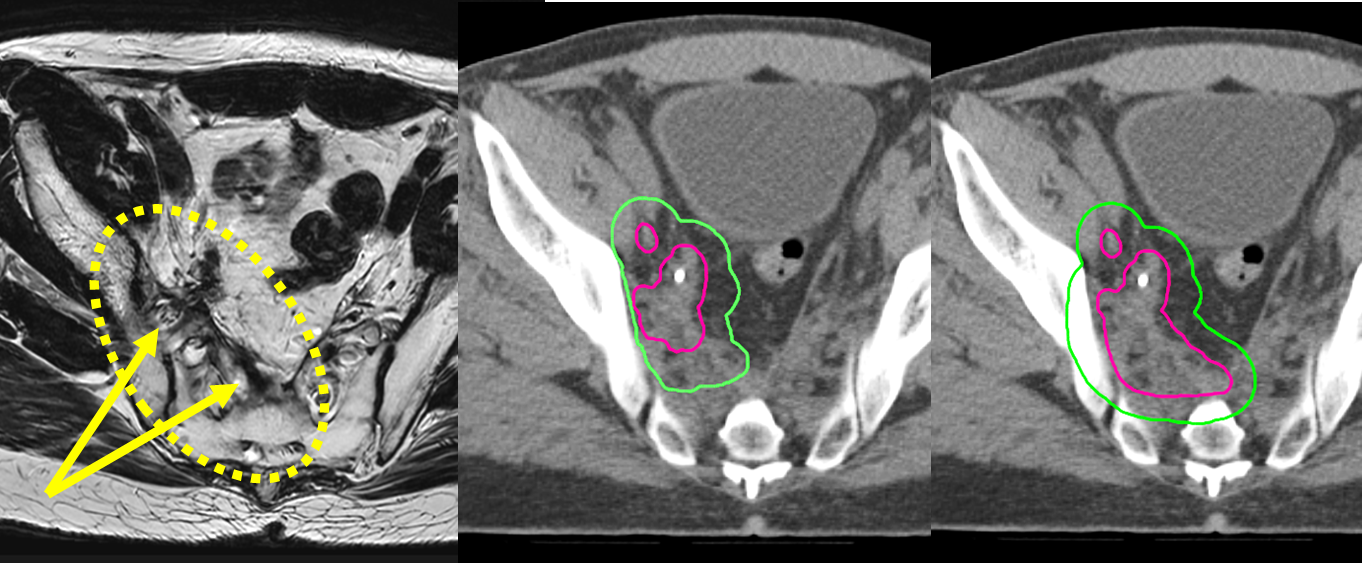


**Figure 7:** Example of a lateral recurrence in which GTV (pink) was extended from before to after QA (middle and right respectively) to encompass complete fibrosis. Subsequently, the CTV (green) was extended and performed editing towards the lateral sidewall was reversed, as there is a risk of undertreatment of surgical resection margins.

Further recommendations in regard to CTV are tailored to patients who are radiotherapy-naïve and to patients undergoing reirradiation.

### Patients undergoing reirradiation

For patients undergoing reirradiation, no elective volumes should be included within the CTV.

In lateral recurrences, elective reirradiation of the complete lateral compartment can be considered.

**Reasoning**: In a (not yet published) retrospective analysis of re-recurrences after LRRC treatment with curative intent, several lateral recurrences re-recurred within the lateral compartment, as shown in figure 8. As the potential toxicity of lateral compartment reirradiation seems less significant than complications arising from surgical resection of the lateral compartment, consensus was reached that elective reirradiation of the lateral compartment can be considered in lateral recurrences.


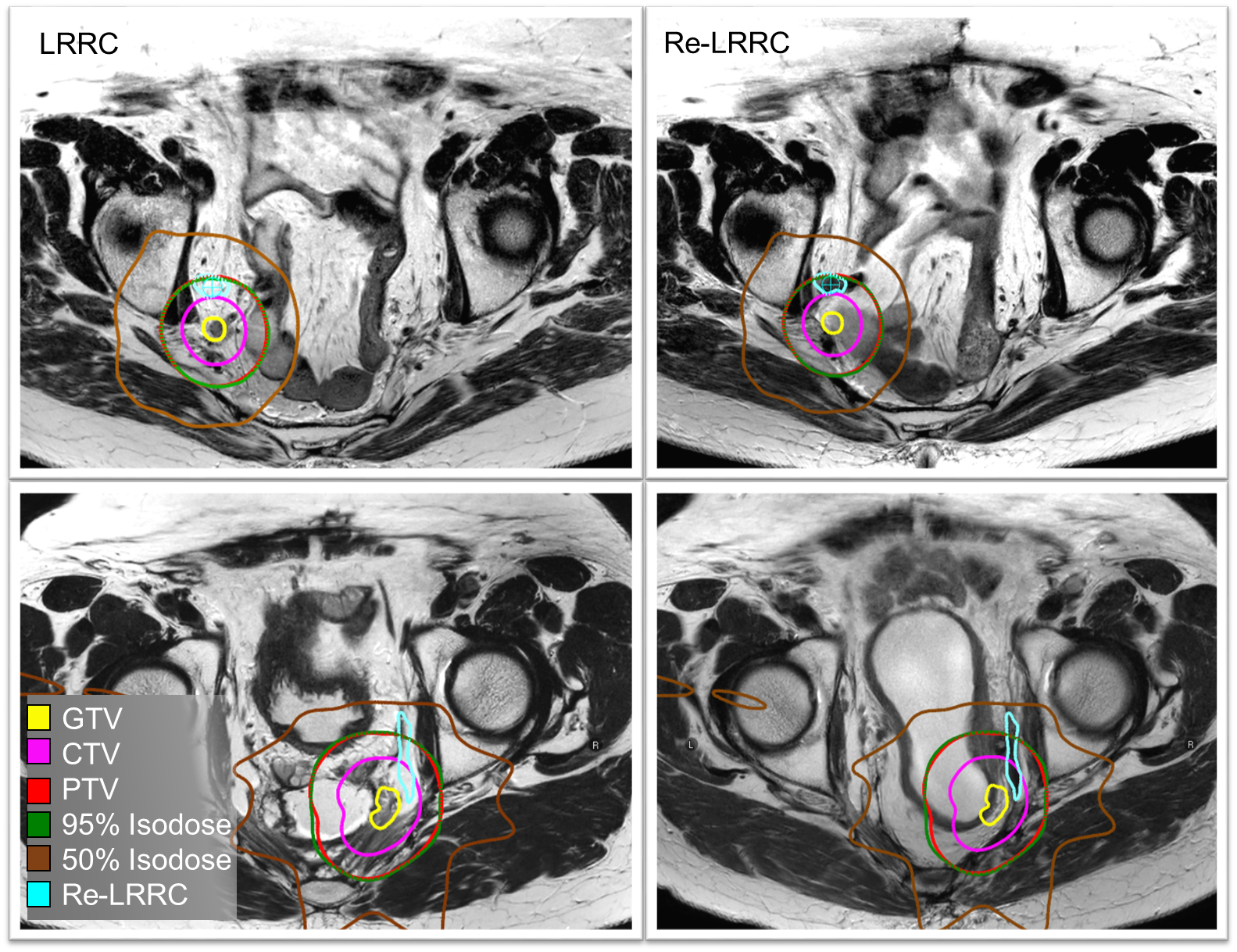


**Figure 8**: First (left) and second recurrence (right) shown on MRI of two individual cases (above and below). First and second recurrence MRIs have been matched to the LRRC planning CT, to be able to visualise the recurrence in the anatomy of the re-recurrence, and vice versa. The re-recurrence is shown in light blue. LRRC with target volumes (GTV yellow, CTV pink, PTV red) and isodose lines (95% green, 50% brown) are also shown. In both cases, re-recurrences developed in the lateral compartment, at the margins of the original PTV.

### RT-naive patients

In general, RT-naive patients should be handled according to the guidelines of Valentini et al.^4^ This leads to the following recommendations:

1. Any remaining mesorectal fat should be included in the CTV, even though exact delineation of the mesorectum may not be possible due to prior surgery.
2. The cranial border of the CTV is the bifurcation of the common iliac arteries into the external and internal iliac artery/sacral promontory, with allowance for national adjustments. The CTV should be extended in the cranial direction if there is a suspicion of the local recurrence, or any (possibly) involved lymph nodes extending beyond this upper limit. In that case, the cranial border should be at least one centimetre above the most cranial GTV.
3. The following lymph nodes should always be included in the CTV;
   1. Mesorectal nodes
   2. Presacral nodes
   3. Internal iliac nodes
   4. Obturator nodes
4. Incorporating the following lymph nodes in the CTV should be considered in case of involvement:
   1. Abdominal presacral lymph nodes
   2. External iliac lymph nodes (in case of unmistakeable involvement).
5. The ischiorectal fossa should be incorporated in the CTV in case of ischiorectal fossa infiltration, or external anal sphincter involvement, or a perineal recurrence.
6. The sphincter complex (if applicable) should be incorporated in the CTV in case of tumour infiltration or perineal recurrences.
7. Incorporating the inguinal lymph nodes in the CTV is not recommended, given the high risk of toxicity and the high risk of morbidity due to surgical resection of inguinal lymph nodes. In case of clear involvement of inguinal lymph nodes, the risk of complications due to additional radiotherapy prior to surgical resection should be weighed appropriately.

Some exceptions do apply:

- In case of distal recurrences and extensive small bowel within the pelvis, decreasing the cranial border to S2/S3 instead of the bifurcation of the common iliac arteries into the external and internal iliac artery/sacral promontory can be considered.
- In case of isolated lymph node recurrences or intraluminal recurrences above S2/S3, it can be considered to not perform irradiation of elective target volumes as described above. It is however recommended to incorporate at least the internal iliac lymph nodes in the CTV until at least two centimeters beyond the GTV caudally.

## Planning target volume (PTV)

This volume ensures coverage of the CTV considering the systematic and random set-up errors, changes over time in the patient geometry and internal organ movement that may occur when delivering a radical course of radiation. Since the required margin is dependent on the local image guidance protocols, the extent of this margin should be determined according to the local policy (between 5mm and 10mm).

# Dose and volume guidelines

All doses are prescribed as target absorbed doses according to International Commission on Radiation Units (ICRU) guidelines.

## Radiotherapy-naive patients

A total dose of 50 Gy in 25 daily fractions of 2.0 Gy per fraction should be delivered. Alternatively, a total dose of 50.4 Gy in 28 fractions can be given.

## Previously irradiated patients

A total dose of 30 Gy in 15 daily fractions of 2.0 Gy per fraction should be delivered.

In patients undergoing reirradiation, it can occur that the PTV is partially located in previously irradiated tissue, whilst the PTV is also partially located within RT-naïve tissue. For example, a patient can present with a distal recurrence, and involved lymph nodes above S1/S2 that have not been previously irradiated. In these cases, it can be considered to give a higher dose (50Gy) to the PTV within RT-naive tissue. In that case, the distal recurrence would be prescribed 30Gy, and the involved lymph nodes would be prescribed 50Gy.

## Target dose constraints

All fields or treatment arcs must be treated during each treatment session. It is conventional to report the dose to the ICRU reference point, the maximum dose to the PTV and the minimum dose to the PTV. The isocentric treatment plan is usually specified to receive 100% with the 95% isodose line encompassing the PTV and no more than +7% and -5% inhomogeneity within the target volume. It is also advised to examine the dose distribution in both coronal and sagittal views to ensure the optimal anatomical arrangement of isodoses around the target volume.

## Organs at risk

The anus, femoral heads, bladder, and small bowel are considered organs at risk (OAR). Delineation of these OARs is mandatory. Further detailed delineation of the anus (i.e., inner and outer sphincter) is strongly recommended but not mandated. OAR contours are defined as below.

- Anus: Inner and outer sphincter, from the level of the anorectal junction. The anorectal junction is easiest recognised on coronal images, at the insertion of the levator ani into the puborectalis (continuous with the external sphincter).
- Small bowel: Small bowel according to hospital specific guidelines, either RTOG, small bowel bag or EMBRACE)
- Bladder: Entire bladder including bladder wall.
- Femoral heads: Contoured to the most inferior extent including the lesser trochanter.

Reliable data on constraints are lacking and highly dependent on the volume and location of the recurrence. Therefore, optimization objectives cannot be given.

For intensity modulated radiotherapy (IMRT) treatment plans, there are no mandated OAR dose-constraints with the priority being target coverage. The order of priority for optimisation in decreasing priority is CTV > PTV > Small bowel > Bladder > Other OAR.

To allow for reliable plan comparisons, the following volume parameters should be reported:

- Volume CTV
- Volume PTV
- Whole body: V5/10/15/20/30/45 Gy
- Bladder: V5/10/15/35/50 Gy

# Treatment planning

Radiation therapy should be delivered with photon energies ≥ 6 MV using a linear accelerator. Equipment of 10 MV or higher is recommended. The use of IMRT or dynamic arc radiotherapy is mandated.

# On treatment verification

Movement of the CTV is known during the course of radiotherapy and is most marked in

the upper rectum. The best available positional verification methods should hence be used which may include electronic portal images compared to digitally reconstructed radiographs (DRRs), or cone-beam CT matching using the planning scan. Adaptive treatment on a MR-Linac is allowed.

Treatment verification should be performed according to local protocol. Acceptable deviations should be assessed according to local policies and the isocentre moved if disagreement is seen in excess of agreed local tolerance levels – usually 5 mm.

# Concomitant chemotherapy

Radiotherapy for radiotherapy-naïve and previously irradiated patients will be combined with capecitabine administered orally in a dose of 825 mg/m2 twice daily on radiotherapy treatment days. In case of unacceptable toxicity of capecitabine during induction chemotherapy (physician’s discretion) Teysuno may be administered orally in a dose of 25mg/m^2^ twice daily on radiotherapy days.

# Minimal requirements for chemoradiotherapy reporting

To enable correct data extraction from the patient file into the electronic case report form, the following issues need to be clearly stated in the patient file:

- Total amount of Gray delivered
- Concomitant chemotherapy agent
- Were any dose reductions necessary?
  - If yes, please state why (i.e., type of toxicity) and specify dose reduction (e.g. stop concomitant chemotherapy)
- Where there any reportable adverse events (AE) during chemoradiotherapy treatment?
  - If yes, please note the severity of the AE according to the National Cancer Institute – Common Terminology Criteria for Adverse Events v5.0

(For the purpose of the PelvEx II study, only AE’s grade ≥3 will be recorded)

# Contact details

*Substantive questions regarding this protocol*Heike Peulen Corrie Marijnen
Radiation Oncologist Radiation Oncologist
Catharina Hospital Eindhoven Netherlands Cancer Institute
E: [heike.peulen@catharinaziekenhuis.nl](mailto:heike.peulen@catharinaziekenhuis.nl) E: [c.marijnen@nki.nl](mailto:c.marijnen@nki.nl)

Maaike Berbee Jeroen Buijsen
Radiation Oncologist Radiation Oncologist
Maastro Maastricht Maastro Maastricht
E: [maaike.berbee@maastro.nl](mailto:maaike.berbee@maastro.nl) E: [jeroen.buijsen@maastro.nl](mailto:jeroen.buijsen@maastro.nl)

Britt Hupkens

Radiation Oncologist

Maastro Maastircht

E: [britt.hupkens@maastro.nl](mailto:britt.hupkens@maastro.nl)

*All other questions*

Davy Creemers

Study coordinator PelvEx II

Catharina Hospital Eindhoven
E: [pelvex2@catharinaziekenhuis.nl](mailto:pelvex2@catharinaziekenhuis.nl)

T: +31 (0)40 2397152

# References

1. Piqeur F, Hupkens BJP, Nordkamp S, et al. Development of a consensus-based delineation guideline for locally recurrent rectal cancer. *Radiotherapy and Oncology*. 2022;177:214-221. doi:10.1016/j.radonc.2022.11.008

2. Piqeur F, Hupkens B, Nordkamp S, et al. PD-0887 Quality assurance of delineation for locally recurrent rectal cancer: PelvEx II data (NCT04389086). *Radiotherapy and Oncology*. 2023;182. doi:10.1016/s0167-8140(23)09012-6

3. Inoue A, Sheedy SP, Wells ML, et al. Rectal cancer pelvic recurrence: imaging patterns and key concepts to guide treatment planning. *Abdominal Radiology*. Published online 2023. doi:10.1007/s00261-022-03746-4

4. Valentini V, Gambacorta MA, Barbaro B, et al. International consensus guidelines on Clinical Target Volume delineation in rectal cancer. *Radiotherapy and Oncology*. 2016;120(2):195-201. doi:10.1016/j.radonc.2016.07.017
